# Role of microRNA carried by small extracellular vesicles in urological tumors

**DOI:** 10.3389/fcell.2023.1192937

**Published:** 2023-06-02

**Authors:** Yiping Mao, Mengting Zhang, Lanfeng Wang, Yukang Lu, Xinyi Hu, Zhiping Chen

**Affiliations:** ^1^ The First School of Clinical Medicine, Gannan Medical University, Ganzhou, China; ^2^ Department of Laboratory Medicine, First Affiliated Hospital of Gannan Medical University, Ganzhou, China; ^3^ Department of Nephrology, First Affiliated Hospital of Gannan Medical University, Ganzhou, China

**Keywords:** small extracelllular vesicles, MicroRNAs, urological tumors, diagnosis, barrett esophagus, therapy

## Abstract

Small extracellular vesicles (sEVs) are minute vesicles secreted by various cells that are capable of transporting cargo, including microRNAs, between donor and recipient cells. MicroRNAs (miRNAs), small non-coding RNAs approximately 22 nucleotides in length, have been implicated in a wide array of biological processes, including those involved in tumorigenesis. Emerging evidence highlights the pivotal role of miRNAs encapsulated in sEVs in both the diagnosis and treatment of urological tumors, with potential implications in epithelial-mesenchymal transition, proliferation, metastasis, angiogenesis, tumor microenvironment and drug resistance. This review provides a brief overview of the biogenesis and functional mechanisms of sEVs and miRNAs, followed by a summarization of recent empirical findings on miRNAs encapsulated in sEVs from three archetypal urologic malignancies: prostate cancer, clear cell renal cell carcinoma, and bladder cancer. We conclude by underscoring the potential of sEV-enclosed miRNAs as both biomarkers and therapeutic targets, with a particular focus on their detection and analysis in biological fluids such as urine, plasma, and serum.

## 1 Introduction

Urologic tumors, primarily composed of prostate cancer (PC), clear cell renal cell carcinoma (ccRCC), and bladder cancer (BC), exhibit a notable increase in incidence with age. From 1990 to 2013, a 2.5-fold global increase was observed in the cumulative number of new cases of kidney, bladder, and prostate cancers. This surge in disease incidence correspondingly resulted in a 1.6-fold increase in overall mortality (Dy et al., 2017). PC stands as one of the three leading causes of cancer-related deaths in men, as well as one of the most frequently diagnosed cancers (Islami et al., 2021). According to 2022 cancer statistics, PC represented 27% of all diagnoses in men. Alarmingly, the proportion of late-stage diagnoses escalated from 3.9% to 8.2% over the previous decade. Despite a stabilization in the decline of the mortality rate, the late-stage incidence rate continued to rise, yielding an average mortality rate of 18.9% (Siegel et al., 2022). BC, being the ninth most common cancer globally, contributes to an estimated 573,000 new cases and 212,000 deaths annually (Sung et al., 2021). Its advanced and metastatic stages are poorly responsive to chemotherapy, leading to suboptimal 5-year survival rates. Furthermore, the cancer-specific mortality rate for BC patients has seen little significant reduction over the past 3 decades (Liu et al., 2022). ccRCC, accounting for nearly 80% of renal cell carcinoma (RCC) subtypes, is the predominant cause of kidney cancer-related deaths (Hsieh et al., 2017). Over the last 2 decades, a yearly 2% increase in RCC incidence has been recorded worldwide. Current RCC cases show low sensitivity to both radiotherapy and chemotherapy, leaving surgery as the primary treatment option. This is particularly true for localized RCC, where surgical intervention remains the sole curative approach (Ljungberg et al., 2022). Despite advancements in treatment leading to an improved 5-year relative survival rate post-diagnosis, the overall prognosis remains poor, especially for patients at advanced stages (Barata and Rini. 2017). Consequently, the urgent medical necessity of identifying reliable diagnostic biomarkers and formulating effective treatment strategies for urologic tumors is apparent.

Small extracellular vesicles (sEVs) are lipid bilayer entities secreted by a wide range of cells that encapsulate a diverse array of biological components, including proteins, lipids, nucleic acids, and other molecular entities (Negahdaripour et al., 2020). As per the MISEV2018 guidelines, these vesicles are characterized by their diminutive size, typically falling below 200 nm, or even 100 nm, in diameter (Théry et al., 2018). Despite the nomenclature ambiguity across different studies, where they may be labeled as exosomes or microvesicles, this paper will consistently use the term “sEVs” to denote these small extracellular vesicles, which are frequently referred to as “exosomes” in scientific literature. Present in virtually all bodily fluids—ranging from blood (Cumba Garcia et al., 2019), urine (He et al., 2019; Wang and Zhang, 2022), saliva (Hofmann et al., 2022), cerebrospinal fluid (Spaull et al., 2019), semen (Wang et al., 2022), amniotic fluid (Sheller-Miller. 2020), malignant ascites (Hu et al., 2019) and pleural effusions (Javadi et al., 2021), bronchoalveolar lavage fluid (Wang et al., 2022) and breast milk (Vaswani et al., 2019; Ramos-Garcia et al., 2023). sEVs perform critical roles in the management and treatment of a multitude of diseases. These include various types of tumors (Zhou et al., 2021), inflammatory diseases (Fan et al., 2022), cardiovascular (Burtenshaw et al., 2022), neurodegenerative (Younas et al., 2022) and renal diseases (Grange and Bussolati. 2022). Their versatile utility spans different stages of cancer treatment, from early diagnosis and screening to detecting minimal residual disease, predicting tumor behavior, designing personalized therapies, and evaluating treatment outcomes and follow-up care (Ghosh et al., 2019). As of today, the primary clinical applications of sEVs encompass drug delivery, biomarkers, therapeutic targets, and anti-cancer vaccines (Kucuk et al., 2021; Fang et al., 2022; Rezaie et al., 2022). MicroRNAs (miRNAs, miRs), constituting a class of small non-coding RNAs approximately 22 nucleotides long, are pivotal in regulating gene expression at the post-transcriptional level (Plawgo and Raczynska. 2022). They exert their regulatory influence by targeting the 3ʹ untranslated region (UTR) of the mRNA of the target gene, consequently modulating protein levels (Bartel. 2009). Owing to their integral function in gene regulation, miRNAs participate in key cellular physiological processes, such as differentiation, proliferation, apoptosis, and development. Furthermore, they play a vital role in the pathogenesis of various diseases, including cancer (Yang et al., 2021; Kousar et al., 2022; Li et al., 2022), diabetes (Zampetaki et al., 2010; He et al., 2021) and cardiovascular diseases (Siasos et al., 2020; Kalayinia et al., 2021).

As integral components of sEVs, miRNAs can be transported from donor to recipient cells, thereby mediating phenotypic alterations (Valadi et al., 2007; O'Brien et al., 2020). These miRNAs, shielded by a lipid bilayer, are resistant to degradation by extracellular nucleases, resulting in heightened stability within body fluids (Munir et al., 2020). A growing body of research reveals a crucial role for sEVs-miRNAs in numerous physiological processes, as well as in the initiation and progression of various diseases. Notably, sEVs-miRNAs exert significant regulatory effects on tumor progression (Sun et al., 2018; Mori et al., 2019). Given their specificity, sensitivity, and stability, sEVs-miRNAs circulating in the humoral fluid are recognized as potentially ideal noninvasive tools for early tumor diagnosis and targeted therapy (Salehi and Sharifi. 2018; Prieto-Vila et al., 2021). This review discusses recent discoveries concerning the role of sEVs-miRNAs in the advancement of urological tumorigenesis and their prospective use as biomarkers and therapeutic targets in urological tumors.

## 2 Biogenesis of sEVs

Extracellular vesicles (EVs) are lipid bilayer-encapsulated particles, produced by various cell types, that are ubiquitously found in physiological fluids. They carry an assortment of biomolecules including proteins, lipids, and nucleic acids (Raposo and Stoorvogel. 2013). Despite a lack of universally accepted classification, the MISEV2018 guidelines offer a categorization based on size, distinguishing between small EVs (sEVs, <200 nm) and medium/large EVs (>200 nm) (Théry et al., 2018). However, a significant number of publications further classify EVs into exosomes (40–150 nm), microvesicles (100–1,000 nm), and apoptotic vesicles (50–1,000 nm) (Willms et al., 2016; Willms et al., 2018). Among these, sEVs, constituted by endosomal-derived and plasma membrane-derived vesicles, are the most abundant in biological fluids (Huang et al., 2021; Qian et al., 2022).

The biogenesis of sEVs initiates with the inward budding of the plasma membrane, leading to the formation of cup-like structures filled with cell surface proteins and extracellular soluble proteins. This invagination yields early sorting endosomes (ESEs) (Kalluri and LeBleu. 2020), which through subsequent maturation, transform into late sorting endosomes (LSEs), and ultimately, multivesicular bodies (MVBs). During the process of MVB formation, multiple intraluminal vesicles (ILVs) are generated, and various cellular proteins, nucleic acids, and lipids are sorted into these vesicles (Abels and Breliefield. 2016). According to existing research, the formation of MVBs is chiefly regulated by the endosomal sorting complex required for transport (ESCRT) (Liu et al., 2021). MVBs may follow one of two pathways: either fusing with autophagosomes and subsequently with lysosomes for degradation or directly interfacing with lysosomes for the same purpose. Alternatively, MVBs may navigate to the plasma membrane through the cytoskeletal and microtubule network, fusing with it to release ILVs into the extracellular space as sEVs (Colombo et al., 2014; van Niel et al., 2018). In addition to endosomal origins, there is compelling evidence that sEVs can also bud directly from the plasma membrane or be sequestered in intracellular plasma membrane–connected compartments (IPMCs) for delayed release. However, this mechanism is constrained by the narrow IPMC neck (Pegtel and Gould, 2019).

## 3 Biogenesis and mechanism of microRNAs

MicroRNAs (miRNAs) are small endogenous non-coding RNAs, typically around 22 nucleotides long, that can modulate gene expression by transcriptional repression or silencing (Lu and Rothenberg, 2018). Our study revealed that the majority of mature miRNA sequences are positioned within the introns of pre-mRNA, as well as within the introns or exons of non-coding RNAs (Saliminejad et al., 2019). The biogenesis of miRNAs primarily involves transcription by RNA polymerase II, which generates primary miRNA (pri-miRNA) stem loops. These stem loops possess a 5′cap structure and can undergo both polyadenylation and splicing (Bushati and Cohen. 2007). The classical pathway of miRNA maturation commences in the nucleus, where a multiprotein microprocessor complex processes the pri-miRNA. This complex primarily consists of the RNase III family nuclease Drosha2 and the double-stranded RNA binding domain (dsRBD) protein DGCR8/Pasha, producing a pre-miRNA with a stem-loop structure of approximately 70 nucleotides (Bushati and Cohen. 2007). This pre-miRNA is recognized by Exportin-5, which facilitates its translocation to the cytoplasm through a Ran-GTP-dependent mechanism (Bohnsack et al., 2004). Once in the cytoplasm, the pre-miRNA is subject to further processing by the RNAase III enzyme Dicer, resulting in the creation of short RNA duplexes. Subsequently, one strand is degraded while the mature miRNA is incorporated into the RNA-induced silencing complex, containing the Argonaute protein. This assembly process results in the mature miRNA being directed to its 3′UTR, binding to the target mRNA via base pairing, which subsequently leads to mRNA degradation or translational inhibition (Kobayashi and Tomari, 2016; Shefler et al., 2019).

## 4 The role of miRNAs carried by sEVs in the development of urological tumors

**FIGURE 1:**
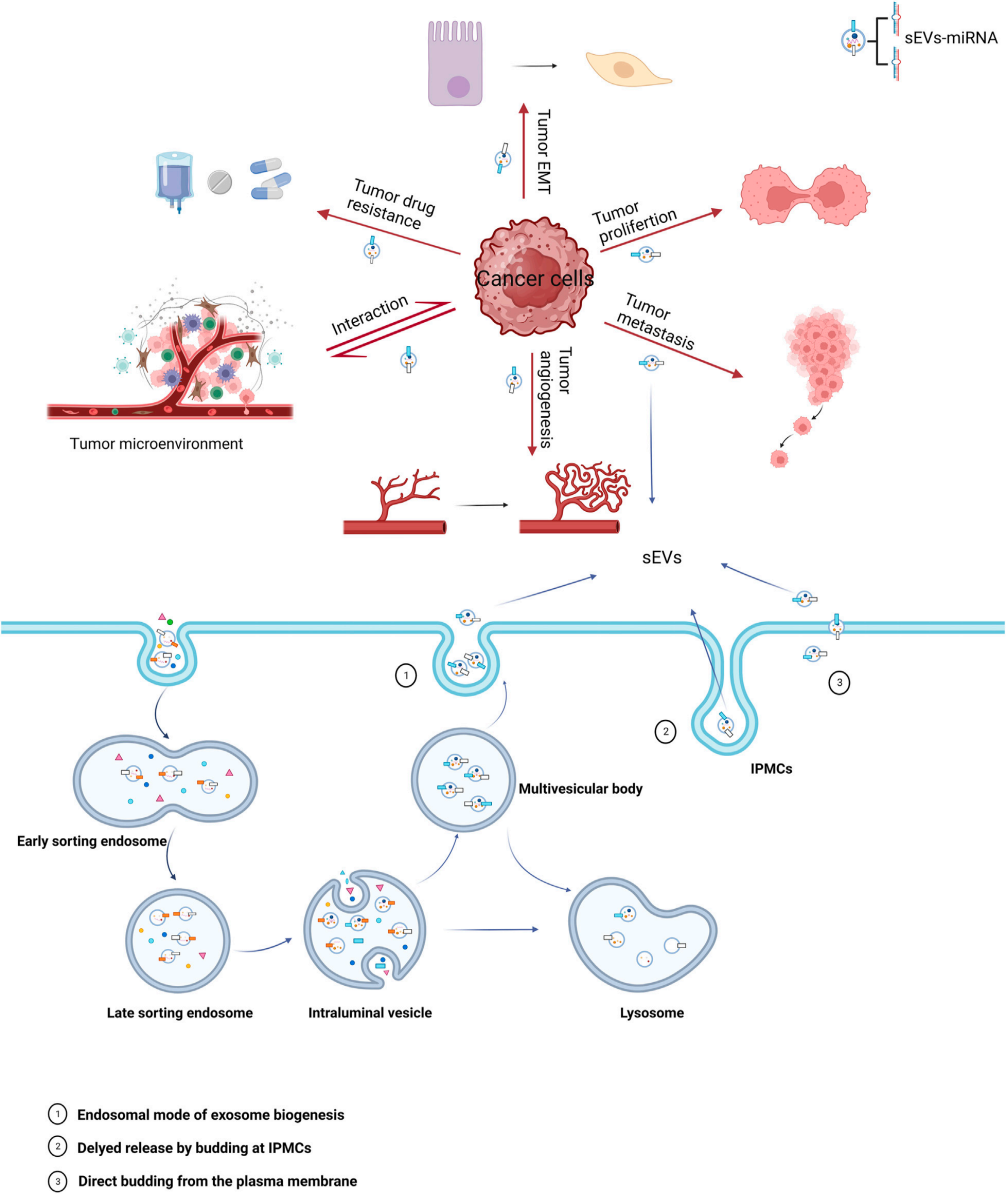
Biogenesis of sEVs and their role in cancer. The miRNAs carried by sEVs in cancer cells play a significant role in all stages of oncogenesis, with major mechanisms including epithelial-mesenchymal transition (EMT), proliferation, metastasis, angiogenesis, tumor microenvironment, and drug resistance.

miRNAs carried by sEVs have been demonstrated to play a significant role in the pathogenesis and progression of urological tumors (Figure 1). Their influence on processes such as invasion, metastasis, angiogenesis, immune evasion, and chemoresistance has been validated by several studies (Huang et al., 2013; Liu et al., 2022; Song et al., 2022) (Table 1).

**TABLE 1:** Role of miRNAs carried by sEVs in the progression of urological tumors.

Cancer types	sEVs-miRNA	Donor cells	Recipient cells	Targets	Application	Ref.
sEVs-miRNA promotes epithelial-mesenchymal transition (EMT) in urological tumors.						
Prostate cancer	miR-217, miR-23b-3p	PC-3, DU145	PC-3, DU145	E-Cadherin, N-Cadherin, Vimentin	Promotes the EMT and regulates PC cell proliferation and invasive ability	Zhou et al. (2020)
Prostate cancer	miR-95	M2-TAM	PC3, DU145	JunB	Promotes PC cell proliferation and invasion and the EMT	Guan et al. (2020)
Bladder cancer	miR-663b	T24, 5637	T24, 5637	ERF	Promotes BC cell proliferation and the EMT	Yin et al. (2020)
Clear cell renal cell carcinoma	miR-181d-5p	CAFs	ACHN, 786-O	RNF43	Promotes ccRCC cell migration and invasion and the EMT	Ding et al. (2022)
Clear cell renal cell carcinoma	miR-15a	ACHN	UMRC-2	BTG2	Promotes ccRCC cell migration and invasion and the EMT	Li et al. (2021)
sEVs-miRNA promotes proliferation and migration in urological tumors.						
Prostate cancer	miR-146a-5p	CAF	LNCaP, DU145	EGFR	Promotes PC cell migration and the EMT	Zhang et al. (2020)
Bladder cancer	miR-21	T24	M0-TAM	PTEN	Promotes BC cell invasion and migration	Lin et al. (2020)
Bladder cancer	miR-217	hBSC	T24, 5367	YAP	Promotes BC cell proliferation and migration	Huang et al. (2021)
Clear cell renal cell carcinoma	miR-155	786-O, Caki-1	786-O, Caki-1	FOXO3	Hypoxia-induced conditions stimulate the proliferation and migration of ccRCC cell	Meng et al. (2021)
Clear cell renal cell carcinoma	miR-155-5p	TAM	ACHN, 786-O	HuR	Hypoxic conditions of TAM promote ccRCC cell proliferation and metastasis.	Gu et al. (2021)
sEVs-miRNA promotes angiogenesis in urological tumors						
Prostate cancer	miR-27a-3p	PC-3	HUVEC	Not yet researched	Promotes angiogenesis	Prigol et al. (2021)
Bladder cancer	miR-93-5p	T24, 5637	HUVEC	Not yet researched	Promotes BC cell proliferation, invasion and angiogenesis	Yuan et al. (2023)
Clear cell renal cell carcinoma	miR-193a-5p	TAM	786-O, Caki-1	TIMP2	Promotes ccRCC cell VM and invasion	Liu et al. (2022)
sEVs-miRNAs promote the microenvironment in urological tumors						
Prostate cancer	miR-1290	CAF	PC3, 22RV1	GSK3β	Promotes PC cell growth and metastasis	Wang et al. (2022)
Prostate cancer	miR-100-5p, miR-21-5p	CSC	WPMY-1	Not yet researched	Promotes tumor growth, survival and proliferation to distant ecological niches	Sánchez et al. (2016)
Prostate cancer	miR-375	LNCaP	hFOB1.19	Not yet researched	Promotes osteoblast activity	Li et al. (2019)
Prostate cancer	miR-1275	PC3	hFOB1.19	SIRT2	Promotes osteoclast proliferation and activity	Zou et al. (2021)
Bladder cancer	miR-186-5p,	T24, SV-HUC-1	NK cell	DAP10	Promotes NK cell dysfunction	Huyan et al. (2022)
Bladder cancer	miR-221-5p	T24, SV-HUC-1	NK cell	CD96, PRF1	Promotes NK cell dysfunction	Huyan et al. (2022)
Clear cell renal cell carcinoma	miR-224-5p	CAF	769-P	Not yet researched	Promotes ccRCC cell proliferation, migration and invasion and inhibits apoptosis	Liu et al. (2021)
Clear cell renal cell carcinoma	miR-19b-3p	CSC	ACHN, 786-O	PTEN	Promotes ccRCC EMT	Li et al. (2019)
Clear cell renal cell carcinoma	miR-142-3p	RCSC	HK2	ERp44	Renal impairment	Wu et al. (2022)
Clear cell renal cell carcinoma	miRNA-21-5p	M2-TAM	ACHN, 786-O	PTEN	Promotes ccRCC metastasis	Zhang et al. (2022)
sEVs-miRNAs promote drug resistance in urological tumors						
Prostate cancer	miR-423-5p	CAF	LN-CaP, 22Rv-1, C4-2	GREM2	Increases resistance of prostate cancer to taxane	Shan et al. (2020)
Prostate cancer	miR-27a	PSC27	PC-3	p53	Mediates chemoresistance in PC-3 cell	Cao et al. (2019)
Bladder cancer	miR-148b-3p	CAF	5637, T24	PTEN	Promotes BC cell the EMT, metastasis and drug resistance	Shan et al. (2021)
Clear cell renal cell carcinoma	miR-31-5p	ACHN, 786-O	ACHN, 786-O	MLH1	Promotes ccRCC cell resistance to sorafenib	He et al. (2020)

### 4.1 Role of epithelial-mesenchymal transition in urological tumors

The epithelial-mesenchymal transition (EMT) is a vital biological process characterized by cells transitioning from an epithelial phenotype to a mesenchymal one. This transition involves a decrease in cell-to-cell adhesion accompanied by an increase in the capability for metastasis and tissue invasion, which are key facilitators of tumor progression and metastasis (Pastushenko and Blanpain. 2019).

In PC, Zhou et al. (2020) examined the impact of sEVs-miR-217 and miR-23b-3p on EMT-related factors (E-calmodulin, N-calmodulin, and Vimentin). A series of *in vitro* and *in vivo* experiments led them to determine that sEVs-miR-217 and miR-23b-3p could regulate EMT in PC cells. Furthermore, these molecules could influence the proliferation and invasive capacity of PC cells through EMT.

In BC, researchers discovered elevated plasma levels of miR-663b carried by sEVs in BC patients compared to a normal control group. It was also discerned that sEVs-miR-663b could foster cell proliferation and EMT by targeting the Ets2-suppressor (ERF) (Yin et al., 2020).

In ccRCC, the Wnt/β-linked protein signaling pathway, a crucial pathway in ccRCC, is modulated by a variety of factors, including sEVs-miRNA (Joosten et al., 2018; Liu et al., 2022). Ding et al. (2022) experimentally demonstrated that sEVs-miR-181d-5p originating from cancer-associated fibroblasts (CAFs) could activate Wnt/β-linked protein signaling in ccRCC cells. This activation occurs by directly repressing the expression of the ring finger 43 (RNF43) protein, subsequently promoting migration, invasion, and EMT of ccRCC cells. In a separate study, sEVs-miR-15a was found to be upregulated in ccRCC cells, thus fostering EMT, and by extension, ccRCC metastasis and growth, by downregulating BTG2 and enhancing PI3K/AKT signaling pathway activity. (Li et al., 2021).

Numerous studies collectively indicate that sEVs-associated miRNAs play a pivotal role in promoting EMT in urological tumors, thereby driving the progression of these tumors. By exploring the regulatory mechanisms of these sEVs-miRNAs on urological tumors, we can enhance our understanding and potentially identify novel therapeutic targets for treating these malignancies.

### 4.2 Implications of sEVs-miRNA in proliferation and migration of urological tumors

Tumorigenic cellular behaviors, encompassing proliferation, invasion, and migration, are driven by a multitude of factors. Among these, sEVs-miRNA emerges as a key player, fostering tumor expansion and metastasis (Liu et al., 2022).

In PC, a unique role of CAFs-derived sEVs has been noted post-androgen deprivation therapy (ADT). It is observed that these CAFs-derived sEVs, enriched with miR-146a-5p, enhance both migration and invasion of EMT and PC cells under ADT. This occurs via modulation of the epidermal growth factor receptor (EGFR)/ERK pathway, thereby contributing to PC metastasis (Zhang et al., 2020).

With regard to BC, research by Lin et al. (2020) demonstrated that BC cell-derived sEVs-miR-21 could boost BC cell invasion and migration. This is achieved by the downregulation of PTEN expression, which subsequently activates the PI3K/AKT-mediated STAT3 signaling pathway in TAMs, leading to the promotion of M2 phenotypic polarization. This is achieved by the downregulation of PTEN expression, which subsequently activates the PI3K/AKT-mediated STAT3 signaling pathway in TAMs, leading to the promotion of M2 phenotypic polarization. Additionally, human bladder mesenchymal stromal cell (hBSC)-derived sEVs-miR-217 mimics have been found to bolster BC cell proliferation and migration while suppressing apoptosis. Conversely, hBSC-derived sEVs-miR-217 inhibitors serve to suppress BC cell proliferation and migration while promoting apoptosis. This regulatory dynamic is mediated through the transcription factor YAP and its target proteins including Cyr61, CTGF, and ANKRD1, which collectively influence BC cell proliferation, migration, and apoptosis (Huang et al., 2021).

In ccRCC, a correlation between tumor progression and the degree of hypoxia, often resultant from rapid tumor growth, is evident (Mennerich et al., 2019). Notably, ccRCC cells under normoxic and hypoxic conditions are found to produce sEVs-miR-155. This results in the hypoxia-induced upregulation of sEVs-miR-155, which directly facilitates ccRCC cell proliferation by suppressing FOXO3 expression (Meng et al., 2021). Furthermore, hypoxic TAM-derived sEVs-miR-155-5p has been reported to enhance RCC cell proliferation and metastasis through the activation of the HuR-dependent IGF1R/AKT/PI3K pathway, thus promoting RCC progression (Gu et al., 2021).

Collectively, these findings underscore the potent influence of sEVs-miRNA in driving the proliferation and metastasis of urological tumors, consequently expediting the progression of urological malignancies.

### 4.3 The role of angiogenesis in urological tumors

Angiogenesis, the formation of new blood vessels, is a crucial component in the study of urological tumors. This process can be visualized and assessed via the tubular network established by human umbilical vein endothelial cells (HUVECs), which faithfully retain the characteristics of vascular endothelial cells. As such, HUVECs present an effective model for the investigation of controlled angiogenesis or neovascularization mechanisms (Park et al., 2006). The connection between angiogenesis and miRNAs in both *in vitro* and *in vivo* environments was first elucidated by Poliseno et al. (2006). Their work demonstrated that the silencing of Dicer and Drosha enzymes in HUVECs diminished tubulogenesis. Since angiogenesis is integral to tumor cell survival, sEVs-miRNAs have been identified as key contributors to angiogenesis in urological tumors. For instance, a study by Prigol et al. (2021) indicated that sEVs from PC-3 cells prompted angiogenic behavior in HUVECs, a finding attributable to the overexpression of miR-27a-3p in PC-3 sEVs. This observation suggests the potential involvement of miR-27a-3p in pro-angiogenic effects. In BC, recent research has revealed that BC cell-derived sEVs containing miR-93-5p significantly enhance cell proliferation, migration, invasion, and angiogenesis. These findings were confirmed through a combination of bioinformatics techniques and comprehensive experimental validations (Yuan et al., 2023). In the case of ccRCC, miR-193a-5p, carried by tumor-associated macrophage (TAM)-derived sEVs, augments vasculogenic mimicry (VM) and cell invasion of ccRCC cells. This occurs through the targeting of the 3′UTR of TIMP2 mRNA in ccRCC cells, inhibiting its translation and consequently promoting angiogenesis (Liu et al., 2022).

### 4.4 Promotion of urological tumor microenvironment

The tumor microenvironment (TME) is a complex network comprised of a multitude of components, including but not limited to, tumor cells, CAFs, endothelial cells, immune cells, and the extracellular matrix (ECM). Additionally, it encompasses non-cellular entities such as sEVs and cytokines (Tan et al., 2020). The intricate interactions between these components, particularly with tumor cells, serve as critical influencers in the progression and development of the tumor (Arneth. 2019).

Within the TME, CAFs represent the most prolific stromal cell type. Of particular interest is the significant role that sEVs derived from CAFs play in tumor development (Nilendu et al., 2018; Sundararajan et al., 2018). For instance, in PC, it has been observed that these CAF-derived sEVs facilitate PC cell migration and invasion. Moreover, they also instigate the upregulation of miR-1290 within the CAF-sEVs. The resulting CAF-sEV-miR-1290 complex promotes PC cell migration, invasion, EMT, and stemness, as evidenced by various cellular and real-time quantitative polymerase chain reaction (RT-qPCR) assays. The underlying mechanism appears to be the inhibition of GSK3β/β-catenin signaling, leading to enhanced PC cell growth and metastasis (Wang et al., 2022). In the context of ccRCC, research by Liu et al. (2021) has revealed a similar pattern. They discovered that CAF-derived exosomes contribute positively to ccRCC cell proliferation, migration, and invasion while exerting an inhibitory effect on apoptosis. These findings were corroborated through cell function, co-culture experiments, and flow cytometry assays. Further, they ascertained that miR-224-5p could be transferred to ccRCC cells via CAF-derived sEVs. Exploring this interaction, they found that overexpression of miR-224-5p led to a significant increase in cell migration and invasion. In conclusion, their research demonstrated that the CAF-sEV-miR-224-5p complex could be internalized by ccRCC cells, subsequently promoting cell proliferation, migration, invasion, and apoptosis inhibition.

Cancer stem cells (CSCs) within the TME exert oncogenic influences, fostering cancer progression, dissemination, and metastasis. Notably, sEVs derived from CSCs echo these functional attributes in neoplastic conditions (Scioli et al., 2021; Liu et al., 2022). In the context of PC, an investigation involving the precipitation of purified exosomes for miRNA extraction and the subsequent comparison of these miRNAs via next-generation sequencing on the Illumina platform was carried out. This analysis revealed six miRNAs with overexpression in CSC-derived exosomes, with miR-100-5p and miR-21-5p being the most profuse. Further bioinformatics scrutiny highlighted that these differentially expressed miRNAs primarily regulate angiogenesis and cellular proliferation, essential functions underpinning tumor growth, survival, and metastatic propagation (Sánchez et al., 2016). Yet, the specific mechanisms underlying these interactions remain elusive, warranting further exploration. In ccRCC, a study by Wang et al. (2019) discerned a markedly higher migratory and invasive potential in CSC-derived exosomes compared to cancer exosomes. This was demonstrated through wound healing, transwell assays, and Western blotting analyses, revealing an impact on the expression of EMT-related genes. This suggests that CSC-sEVs may hasten EMT in ccRCC cells. Further examination using quantitative real-time polymerase chain reaction (qRT-PCR) to detect and manipulate the expression of miR-19b-3p within CSC-sEVs via lentivirus revealed that CSC-sEV-miR-19b-3p levels were significantly elevated compared to those in cancer exosomes. The influence of CSC-derived exosomes was curtailed following the knockdown of miR-19b-3p, implying a mediating role for this miRNA in CSC-exosome action. Subsequent Western blotting and luciferase activity measurements affirmed that CSC-sEV-miR-19b-3p could stimulate EMT by suppressing PTEN expression, thus enhancing the metastatic propensity of ccRCC cells. Similarly, Wu et al. (2022) discovered that renal CSC (RCSC)-derived sEVs induced apoptosis and endoplasmic reticulum stress in kidney cells, as demonstrated by FCM assay, TUNEL staining, and Western blotting. Following local injection of RCSC-sEVs in mice, histological and immunohistochemical analyses deduced that RCSC-sEV-miR-142-3p could impair renal function. Concurrently, dual luciferase activity measurements and Western blotting analyses established that miR-142-3p, derived from kidney CSC-sEVs, could be expressed in renal cells by interfering with ERp44. This led to the activation of the PERK-CHOP pathway, subsequently inducing endoplasmic reticulum stress and apoptosis in renal cells, culminating in renal impairment. Nevertheless, this complex process of renal impairment mediated by RCSC-sEVs necessitates further elucidation through extensive research.

In the TME, numerous constituents contribute significantly to the evolution of urologic tumors. Notably, in PC, osteoblastic bone metastases frequently emerge, underscoring the crucial role of osteoblast activity regulation in managing PC metastases (Borel et al., 2020). Recent studies have identified sEVs-miRNAs as crucial mediators of the interface between PC cells and the bone metastasis microenvironment. Among these, sEVs-miR-375 and miR-1275 have drawn considerable attention (Li et al., 2019; Zou et al., 2021). Li et al. (2019) demonstrated that sEVs-miR-375, derived from LNCaP cells, significantly enhanced osteoblast activity. This effect was confirmed through a series of experiments, including transfection of osteoblasts with miR-375 mimics, the subsequent evaluation of alkaline phosphatase activity, extracellular matrix mineralization, and the expression of osteoblast activity-related marker genes. In another investigation, PC3 cell-derived sEVs-miR-1275 was found to promote osteoblast proliferation and activity by modulating the SIRT2/Runx2 signaling pathway. Techniques such as ultracentrifugation, qRT-PCR, and CCK-8 assays were employed to isolate exosomes from PC3-derived conditioned medium, supporting the role of sEVs-miR-1275 as a vital enhancer of osteoblast activity (Zou et al., 2021). Contrastingly, in BC, BC cell-secreted sEVs-miR-186-5p and miR-221-5p have been observed to disrupt mRNA stability in natural killer (NK) cells. They achieve this by targeting the DAP10 and CD96, and perforin genes (PRF1), respectively, in NK cells. The overall result is a decline in NK cell cytotoxicity against target cells and an impairment in NK cell production. (Huyan et al., 2022). In the context of ccRCC, Zhang et al. (2022) discovered that M2 macrophage-derived sEVs-miRNA-21-5p could downregulate a tumor suppressor and activate the Akt signaling pathway to spur ccRCC cell metastasis. This was accomplished by targeting a specific sequence in the PTEN-3′UTR.

Collectively, these findings underscore the dynamic interplay between urological tumors and various TME components mediated by sEVs-miRNAs, ultimately fostering urological tumor progression. Nonetheless, the necessity for additional evidence detailing the effects of sEVs-miRNAs interacting with TME components remains.

### 4.5 Promotion of drug resistance in urological tumors

Drug resistance presents a significant obstacle in tumor therapy, emerging as a major hurdle in its efficacy. This resistance in tumor cells primarily originates from either intrinsic or extrinsic factors. Intrinsic drug resistance stems from genetic or phenotypic modifications within the tumor cells. Conversely, extrinsic drug resistance arises from the tumor’s interaction with its surrounding microenvironment (Steinbichler et al., 2019).

In PC, Shan et al. (2020) demonstrated through a series of studies that the promotion of chemoresistance in PC is mediated by CAF-derived sEVs carrying miR-423-5p, which targets GREM2. Intriguingly, sEVs-miR-423-5p was found to inhibit the activity of the TGF-β pathway, thus inducing resistance in PC cells. *In vivo* analysis demonstrated that miR-423-5p increased the resistance of PC cells to paclitaxel. These studies eventually led to the conclusion that CAF-secreted sEVs-miR-423-5p inhibit GREM2 via the TGF-β pathway, thus increasing resistance to paclitaxel and fostering PC chemoresistance. Furthermore, sEVs-derived miR-27a in PSC27 cells enhances PC chemotherapy resistance by downregulating P53 gene expression (Cao et al., 2019).

In BC, CAF-derived sEVs-miR-148b-3p curbs apoptosis, encourages EMT, metastasis, and drug resistance in BC cells. PTEN was identified as a target of miR-148b-3p. Interestingly, when sEVs-miR-148b-3p is downregulated, PTEN is upregulated, inhibiting EMT, metastasis, and drug resistance in BC cells through the Wnt/β-catenin pathway (Shan et al., 2021).

In ccRCC, He et al. (2020) discovered that miR-31-5p in EV could transfer resistance information from sorafenib-resistant ccRCC cells to sensitive ones. This miR-31-5p was found to promote resistance to sorafenib in ccRCC cells both *in vitro* and *in vivo* by downregulating the MHL1 gene. Elevated miR-31-5p levels were also observed in plasma EV from sorafenib-resistant ccRCC patients.

These investigations highlight the potential mechanisms by which sEVs-miRNA may impair drug efficacy, providing avenues to enhance urological tumor treatment. However, some findings appear to be derived from cellular studies based on inferential evidence and lack rigorous experimental validation. Hence, more robust and standardized experiments are crucial for further substantiation of these findings.

## 5 Diagnostic and prognostic potential of sEVs-carried miRNAs in urological tumors

Early diagnosis and accurate prognosis of tumors are instrumental to the success of cancer treatments. The analysis of miRNAs carried by sEVs has shown substantial potential as a biomarker for early diagnosis and prognosis across a spectrum of cancers (Sun et al., 2018; Xiao et al., 2020; Pinzani et al., 2021) (Table 2).

**TABLE 2:** miRNAs carried by sEVs as potential biomarkers for urological tumors.

Cancer type	sEVs-miRNA	Clinical sample	Up/Downregulation	Cite
Prostate cancer	miR-16, miR-195	Urine	Downregulated	Borkowetz et al. (2020)
	miR-310a	Urine	Upregulated	Hasanoğlu et al. (2021)
	miR-21, miR-451, miR-636	Urine	Upregulated	Shin et al. (2021)
	miR-30b-3p, miR-126-3p	Urine	Upregulated	Matsuzaki et al. (2021)
	miR-375, miR-574-3p	Urine	Upregulated	Lee et al. (2018)
	miR-125a-5p	Plasma	Downregulated	Li et al. (2020)
	miR-141-5p	Plasma	Upregulated	Li et al. (2020)
	miR-10a-5p, miR-29b-3p	Plasma	Upregulated	Worst et al. (2019)
	miR-423-3p	Plasma	Upregulated	Guo et al. (2020)
	miR-654-3p, miR-379-5p	Serum	Upregulated	Yu et al. (2018)
	miR-181a-5p	Serum	Upregulated	Wang et al. (2021)
	miR-1246	Serum	Upregulated	Bhagirath et al. (2018)
	miR-142-3p, miR-142-5p, miR-223 -3p	Semen	Upregulated	Barceló et al. (2019)
Bladder cancer	miR -146b-5p	Urine	Upregulated	Baumgart et al. (2019)
	miR-96-5p, miR-183-5p	Urine	Upregulated	El-Shal et al. (2021)
	miR-21-5p	Urine	Upregulated	Matsuzaki et al. (2017)
	miR-93-5p, miR-516a-5p	Urine	Upregulated	Lin et al. (2021)
	miR-375, miR-146a	Urine	Upregulated	Andreu et al. (2017)
	miR-4669, miR-4298	Plasma	Downregulated	Yan et al. (2020)
	miR-4644	Plasma	Upregulated	Yan et al. (2020)
	miR-663b	Plasma	Upregulated	Yin et al. (2020)
Clear cell renal cell carcinoma	miR-497, miR-663b	Urine	Downregulated	Song et al. (2019)
	miR-30c-5p	Urine	Upregulated	Kurahashi et al. (2019)
	miR-92a-1-5p	Plasma	Downregulated	Xiao et al. (2020)
	miR-1293	Plasma	Downregulated	Dias et al. (2020)
	miR-301a-3p	Plasma	Upregulated	Dias et al. (2020)
	miR-149-3p, miR-424-3p	Plasma	Upregulated	Xiao et al. (2020)
	miR-210, miR-1233	Serum	Upregulated	Zhang et al. (2018)
	miR-4525	Serum	Upregulated	Muramatsu-Maekawa et al. (2021)

Extensive research suggests that sEV-carried miRNAs could serve as potential biomarkers for urological tumors (Zeuschner et al., 2020). An experimental analysis of miRNA detection in the urine of prostate cancer patients with elevated serum prostate-specific antigen (PSA) levels identified sEV-miR-30b-3p and miR-126-3p as potential PC biomarkers (Matsuzaki et al., 2021). Additionally, miRNAs conveyed by sEVs in semen may have considerable diagnostic utility. Three miRNAs in particular, miR-142-3p, miR-142-5p, and miR-223-3p, were detected in semen sEVs and found to be overexpressed in patients with both malignant and benign prostate tumors compared to healthy controls. The combination of these three miRNAs with blood PSA concentrations could aid in distinguishing benign from malignant tumors (Barceló et al., 2019). A separate study concluded that sEVs-miR-221-3p outperformed serum PSA levels in differentiating prostate cancer from BPH. This was determined by evaluating sEVs-miR-21, miR-141, and miR-221 isolated from plasma and comparing these to serum PSA levels (Kim et al., 2021). In the realm of prostate cancer treatment, serum sEVs-miR-654-3p and miR-379-5p have shown potential as noninvasive biomarkers for predicting the efficacy of carbon ion radiotherapy (CIRT) in PC patients undergoing this treatment (Yu et al., 2018).

In the case of BC, recent findings have highlighted the potential of urinary sEVs-miRNA-96-5p and miRNA-183-5p as promising diagnostic biomarkers. These two miRNAs have demonstrated robust sensitivity and specificity in distinguishing BC. Specifically, miR-96-5p showed a sensitivity and specificity of 80.4% and 91.8%, respectively, while the corresponding values for miR-183-5p were 78.4% and 81.6%. Importantly, when these miRNAs were used in combination for BC diagnosis, the sensitivity increased to 88.2% and the specificity to 87.8%, further attesting to their diagnostic potential (El-Shal et al., 2021).

For ccRCC, one study identified significant disparities in miR-30c-5p expression levels within urinary sEVs. The researchers compared patients with early-stage ccRCC and healthy control individuals. For ccRCC diagnosis, the urinary sEVs-miR-30c-5p levels demonstrated a sensitivity of 68.57% and an impressive specificity reaching 100% (Song et al., 2019). A further experimental analysis compared sEVs-miRNA expression in plasma from ccRCC patients with localized disease (both pre- and post-surgery) to those with metastatic disease. This study revealed a consistent decreasing trend in the expression of sEVs-mir-301a-3p among patients who had undergone surgery, in contrast to a significant increase in the metastatic group. These findings suggest that sEVs-mir-301a-3p may assume a crucial role in the metastatic process, and it may hold promise as a prognostic biomarker (Dias et al., 2020).

Taken together, it is easy to find that miRNAs of sEVs origin can be potential biomarkers for urological tumors due to their specificity, wide source, and stability.

## 6 Therapeutic applications of sEVs

Recent advancements in the field of sEV research have uncovered the multifaceted roles of sEVs in cancer therapy. sEVs are not only ideal noninvasive biomarkers for disease diagnosis but also effective drug delivery vehicles for diverse cancer therapies. Their membrane permeability enables them to traverse biological barriers, including the blood-brain barrier (Zhuang et al., 2011; Patil et al., 2020). A variety of cell types, such as immune cells, mesenchymal stem cells (MSCs), and cancer cells, can serve as sources for sEV-based drug delivery systems (Walker et al., 2019). Given the potent tumor-targeting capability, low immunogenicity, high tolerance, and nanoparticle properties of bioengineered MSC-derived sEVs, they are frequently employed as carriers for the delivery of various functional RNAs, natural compounds, or chemotherapeutic agents in oncology treatment (Weng et al., 2021). Numerous experimental studies have delineated methods for loading therapeutic molecules into sEVs. These include pre-loading, post-loading, and the creation of artificial structures mimicking natural sEVs (Sil et al., 2020). Apart from their role in drug delivery, sEVs also show substantial promise in cancer immunotherapy. sEVs derived from a range of cell types, including B cells, dendritic cells (DCs), macrophages, cancer cells, and normal cells, have potential use in cancer immunotherapy. The hope is that these could eventually be developed into tumor vaccines, adding another powerful tool to the arsenal of cancer therapies (Thakur et al., 2022).

Experimental studies have underscored the therapeutic potential of miRNAs carried by sEVs for urologic tumors (Linxweiler and Junker, 2020) (Table 3). In the context of PC, sEVs-miRNAs such as miR-320a-3p and miR-186-5p have been identified through next-generation sequencing (NGS) analysis. These miRNAs have potential as grading tools for patients with ISUP grade 1, 2, and 3 prostate cancer, suggesting that regular monitoring of urinary sEVs-miRNAs in PC patients could enhance therapeutic strategies (Ramirez-Garrastacho et al., 2022). Recent reports also highlight that sEVs-miR-26a and sEVs-miR-1246 can impede tumor development through the inhibition of EMT in PC. Specifically, sEVs-miR-26a may suppress the EMT process in PC by modulating the expression of EMT-related factors (Wang et al., 2019). On the other hand, sEVs-miR-1246 may inhibit EMT by repressing PC mesenchymal genes, thereby exerting multifaceted effects on cell proliferation, apoptosis, invasion, and migration (Bhagirath et al., 2018). MSC-derived sEVs have been utilized to deliver miR-let-7c to Castration-resistant prostate cancer (CRPC), impeding cell proliferation and migration and providing a targeted approach to CRPC (Kurniawati et al., 2022).

**TABLE 3:** Therapeutic potential of miRNAs carried by sEVs for urological tumors.

Cancer types	sEVs-miRNA	Clinical sample	Biological function	Cite
Prostate cancer	miR-654-3p、miR-379-5p	Serum	Predicting the efficacy of CIRT for PC	Yu et al. (2018)
	miR-1246	Serum	Inhibition of PC metastasis and tumor growth	Bhagirath et al. (2018)
	miR-99b-5p	HBMSCs	Inhibition of PC progression through downregulation of insulin-like growth factor 1 receptor (IGF1R)	Jiang et al. (2022)
	miR-let-7c	HBMSCs	Inhibition of PC cell proliferation and migration	Kurniawati et al. (2022)
	miR-205	HBMSCs	Inhibits PC cell proliferation, invasion and migration and enhances apoptosis by targeting RHPN2	Jiang et al. (2019)
	miR-146a-5p	CAFs	Inhibition of migration and invasion of EMT and PC cells through regulation of the EGFR/ERK pathway	Zhang et al. (2020)
	miR-26a	PC cells	Inhibition of PC metastasis and tumor growth	Wang et al. (2019)
Bladder cancer	miR-133b	Serum	Inhibition of BC cell proliferation and induction of apoptosis by targeting DUSP1	Cai et al. (2020)
	miR-9-3p	BMSC	Inhibits BC cell viability, migration and invasion via ESM1 downregulation; induces apoptosis	Cai et al. (2019)
	miR-139-5p	BMSC	Inhibits the proliferation, migration and invasion of BC cells by regulating the KIF3A/p21 axis, while inducing apoptosis	Xiang et al. (2022)
	miR-139-5p	hUCMSCs	Inhibition of BC cell proliferation by targeting and decreasing the expression of PRC1	Jia et al. (2021)
	miR-138-5p	MSCs	Inhibited migration, invasion and proliferation of BC cells	Liu et al. (2022)
	miR-375-3p	BC cells	Inhibits the proliferation and migration of BC cells and promotes apoptosis	Li et al. (2020)
Clear cell renal cell carcinoma	miR-1	Serum	Inhibition of ccRCC cell proliferation, migration and invasion	Yoshino et al. (2022)
	miR-549a	ccRCC cells	Silencing of HIF1α protein in vascular endothelial cells reduces angiogenesis and endothelial cell migration	Xuan et al. (2021)

In BC, sEVs-miR-375-3p, bone marrow mesenchymal stem cell (BMSC)-derived sEVs-miR-139-5P, and sEVs-miR-9-3p have been found to inhibit the progression of BC (Cai et al., 2019; Li et al., 2020; Xiang et al., 2022). sEVs-miR-375-3p can obstruct the expression of the BC cell growth gene FZD8, thereby inhibiting the Wnt/β-catenin pathway and downstream molecules Cyclin D1 and c-Myc, suppressing proliferation and metastasis while promoting BC cell apoptosis (Li et al., 2020). BMSC-derived sEVs can transfer miR-139-5p into bladder cancer cells, inhibiting proliferation, migration, and invasion while inducing apoptosis. This effect is mediated by BMSC-sEVs-miR-139-5p activating p21 through KIF3A targeting and inhibition, thereby limiting tumorigenesis and lung metastasis in bladder cancer cells (Cai et al., 2019). Human umbilical cord mesenchymal stem cells (hUCMSCs)-derived sEVs-miR-9-3p exert anti-tumor effects by downregulating the tumor promoter gene endothelial cell-specific molecule-1 (ESM-1), leading to reduced BC cell viability, migration, invasion, and enhanced apoptosis (Xiang et al., 2022). Conversely, MSC-derived sEVs-miR-139-5p inhibits BC development by targeting and downregulating cytokinesis 1 (PRC1), thereby suppressing BC cell proliferation and subsequent BC progression (Jia et al., 2021).

In ccRCC, recent findings have demonstrated the potential therapeutic efficacy of sEVs-miR-1 and sEVs-miR-549a (Xuan et al., 2021; Yoshino et al., 2022). sEVs-miR-1 exerts a substantial inhibitory effect on ccRCC cells, curbing cell proliferation, migration, and invasion (Yoshino et al., 2022). Meanwhile, sEVs-miR-549a attenuates angiogenesis and endothelial cell migration by suppressing HIF1α protein in vascular endothelial cells. However, in the context of TKI-resistant renal cancer, which secretes lower levels of sEVs-miR-549a, the inhibition of HIF1α is reduced, thereby facilitating vascular permeability and angiogenesis, which in turn promotes tumor metastasis (Xuan et al., 2021).

Three distinct sets of target genes have been identified to illustrate the involvement of sEVs-miRNAs in urological cancers. These differentially expressed sEVs-miRNAs, either individually or collectively, hold promise as biomarkers. They could play a vital role in various aspects of cancer management, including tumor staging, early diagnosis, progression monitoring, prognostic assessment, and treatment response evaluation. Furthermore, they may also serve as potent therapeutic agents. Despite these promising developments, research in this field remains in its infancy, with many unexplored avenues. Further investigations are necessary to enhance our understanding of sEVs-miRNA-based therapies and their application in the treatment of patients with urological tumors.

## 7 Conclusion

In recent years, sEVs and miRNAs have emerged as an active research Frontier, although their implementation within research methodologies is still relatively nascent. Given the ubiquity of sEVs in various body fluids and their capacity to transport miRNAs to target cells, these entities hold significant promise in cancer research. In the context of urological tumors, the composition of sEV-carried miRNAs within biological fluids differs markedly. Current studies suggest these differential miRNAs can significantly influence cell EMT, proliferation, migration, angiogenesis, TME, and drug resistance. They can exert their influence through the activation or suppression of various regulatory mechanisms *in vivo*. As we move forward, these sEV-carried miRNAs could potentially be harnessed for early detection, diagnosis, prognosis prediction, and treatment efficacy assessment of urological tumors. They offer a promising, noninvasive alternative to biopsy for monitoring recurrence and individual responses to therapy. Moreover, the elucidation of their regulatory pathways could provide novel therapeutic targets to better inhibit tumor progression.

Despite significant strides in the study of sEV-carried miRNAs in urological tumors, several challenges warrant attention. Firstly, the molecular mechanisms underlying sEV generation and their biological roles in tumor progression remain somewhat elusive, necessitating further experimental studies. Secondly, the potential of sEV-carried miRNAs as reliable biomarkers and therapeutic targets for urological tumors remains to be fully explored. Lastly, to guarantee the clinical safety and efficacy of sEV-carried miRNAs as therapeutic agents, large-scale stratification studies are needed to ensure reproducible results. By addressing these challenges, we can strive to translate the diagnostic and therapeutic potential of sEVs into clinical reality.

## Figures and Tables

**Table:** 

**ADT**	Androgen deprivation therapy
**BC**	Bladder cancer
**BMSC**	Bone marrow mesenchymal stem cell
**ccRCC**	Clear cell renal cell carcinoma
**CAFs**	Cancer-associated fibroblasts
**CSCs**	Cancer stem cells
**CIRT**	Carbon ion radiotherapy
**CRPC**	Castration-resistant prostate cancer
**DUSP1**	Dual specific phosphatase −1
**EVs**	Extracellular vesicles
**ESEs**	Early sorting endosomes
**EMT**	Epithelial-mesenchymal transition
**ERF**	Ets2-suppressor
**HUVECs**	Human umbilical vein endothelial cells
**hUCMSCs**	Human umbilical cord mesenchymal stem cells
**HBMSCs**	Human bone marrow mesenchymal stem cells
**ILVs**	Intraluminal vesicles
**IPMCs**	Intracellular plasma membrane–connected compartments
**LSEs**	Late sorting endosomes
**MVBs**	Multivesicular bodies
**MSCs**	Mesenchymal stem cells
**PSA**	Prostate-specific antigen
**pri-miRNA**	Primary miRNA
**PC**	Prostate cancer
**PRC1**	Polycomb repressor complex 1
**RCC**	Renal cell carcinoma
**qRT-PCR**	Quantitative real-time polymerase chain reaction
**RT-qPCR**	Real-time quantitative polymerase chain reaction
**RNF43**	Ring finger 43
**RCSC**	Renal cancer stem cell
**sEVs**	Small extracellular vesicles
**TAM**	Tumor-associated macrophage
**TME**	Tumor microenvironment
**UTR**	Untranslated region
**VM**	Vasculogenic mimicry
